# ﻿The second species of the genus *Ivoria* Kontschán, 2019: description of *Ivoriaalourouai* sp. nov. from Ivory Coast (Acari, Mesostigmata, Urodinychidae)

**DOI:** 10.3897/zookeys.1082.79011

**Published:** 2022-01-18

**Authors:** Jenő Kontschán, Sergey G. Ermilov

**Affiliations:** 1 Plant Protection Institute, Centre for Agricultural Research, ELKH, H-1525 Budapest, PO Box 102, Hungary Plant Protection Institute, Centre for Agricultural Research Budapest Hungary; 2 Institute of Environmental and Agricultural Biology (X-BIO), Tyumen State University, Semakova Str. 10, 625003 Tyumen, Russia Tyumen State University Tyumen Russia

**Keywords:** New species, soil-inhabiting mite, taxonomy, Uropodina, West Africa

## Abstract

A new *Ivoria* species (*Ivoriaalourouai***sp. nov.**) is described from Ivory Coast based on five females. The new species differs from the previously described congener (*Ivoriataienesis* Kontschán, 2019) based on the shape of female genital shield, dorsal setae, centro-caudal part of the marginal shield and peritremes.

## ﻿Introduction

Uropodina are a very diverse group of soil-inhabiting mites, especially in tropical rainforests (Lindquist et al. 2009). Despite this high diversity, these mites remain poorly investigated in many tropical countries, like Ivory Coast, from where only nine species have been reported from the genera *Trichouropoda* Berlese, 1916 sensu lato, *Uroobovella* Berlese, 1903 sensu lato (Wiśniewski 1993) and *Rotundabaloghia* Hirschmann, 1975 (Kontschán 2009), *Ivoria* Kontschán, 2019, *Mahnertellina* Kontschán, 2020 and *Origmatrachis* Hirschmann, 1979 (Kontschán 2019, 2020a, b).

The genus *Ivoria* was described from Taï National Park in Ivory Coast (Kontschán 2019), which contains one of the largest primary rainforests in West Africa. In recent years, the first author spent numerous weeks in the Natural History Museum of Geneva to study the Uropodina mite diversity of the tropical soils. During the investigation of the West African soil samples, the second species of *Ivoria* was discovered from Taï National Park in a disturbed area close to the village Dropleu. This new species is described in this paper.

## ﻿Materials and methods

Specimens were cleared in lactic acid for a week and investigated with a Leica 1000 scientific microscope with a drawing tube. The photos were taken with a Keyence 5000 digital microscope. Specimens examined were stored in 70% ethanol and deposited in the Natural History Museum, Geneva (NHMG). Measurements are given in micrometers (μm).

### ﻿Abbreviations

Setae and pores: **st1–5** sternal setae; **h1–h4** hypostomal setae; **p** pores.

## ﻿Taxonomy

### ﻿Suborder Uropodina Kramer, 1881

#### Family Urodinychidae Berlese, 1917

##### ﻿Genus Ivoria

Taxon classificationAnimaliaMesostigmataUrodinychidae

Kontschán, 2019

037EE0E7-7D09-56B3-82E9-208C64689095

Ivoria Kontschán 2019: 1024.

###### Type species.

*Ivoriataiensis* Kontschán, 2019

###### Diagnosis.

Idiosoma subpentagonal, dorsally domed, marginal and dorsal shields fused anteriorly. All dorsal setae short, with pilose or serrate distal margins. Five pairs of sternal setae smooth or pilose. Genital shield of female subtriangular. Peritreme L-shaped or hook-shaped. Tritosternum with vase-like base, apically serrate, laciniae subdivided into two pairs of short lateral and one pair of long central branches. Hypostomal setae *h1* robust, basally with lateral teeth, *h2*, *h3*, and *h4* narrow and marginally serrate. Palptrochanter setae *v1* robust and serrate, *v2* situated on small protuberance and divided into a short smooth and a long, basally serrate and apically pilose branches. Corniculi small and horn-like, situated at posterior level of *h2*. Internal malae long and smooth. Chelicerae large and robust with internal sclerotized nodes, movable digit shorter than fixed digit, both digits bearing a large central tooth in addition to smaller subapical teeth. Leg I without ambulacral claws; majority of leg setae marginally pilose.

###### Remarks.

The robust and large chelicerae occur only in some genera within the Uropodina. The following genera *Baloghjkaszabia* Hirschmann, 1973, *Kaszabjbaloghia* Hirschmann, 1973, *Wernerhirschmannia* Hiramatsu, 1983, *Multidenturopoda* Wiśniewski & Hirschmann, 1991, *Bloszykiella* Kontschán, 2010, *Editella* Kontschán, 2011 and *Jedediella* Kontschán & Starý, 2012 have large and robust chelicerae; the most important differences among them are summarized in Kontschán (2019: table 1). Unfortunately, families of Uropodina are not well defined, and the classification system of Uropodina is confusing, so it is questionable which genera belong to the family Urodinychidae. The chelicerae of the other members (like species of the genus *Uroobovella* sensu lato) in the family Urodinychidae are small, narrow, and usually have a shorter or longer apical prolongation on the fixed digit.

##### ﻿ Ivoria alourouai sp. nov.

Taxon classificationAnimaliaMesostigmataUrodinychidae

46FAB210-3C0C-58A6-B2D5-3E0B6ECB3B25

http://zoobank.org/B83D5B76-2A95-47D8-A1C1-A52AB3DA7964

**Figure 1.**
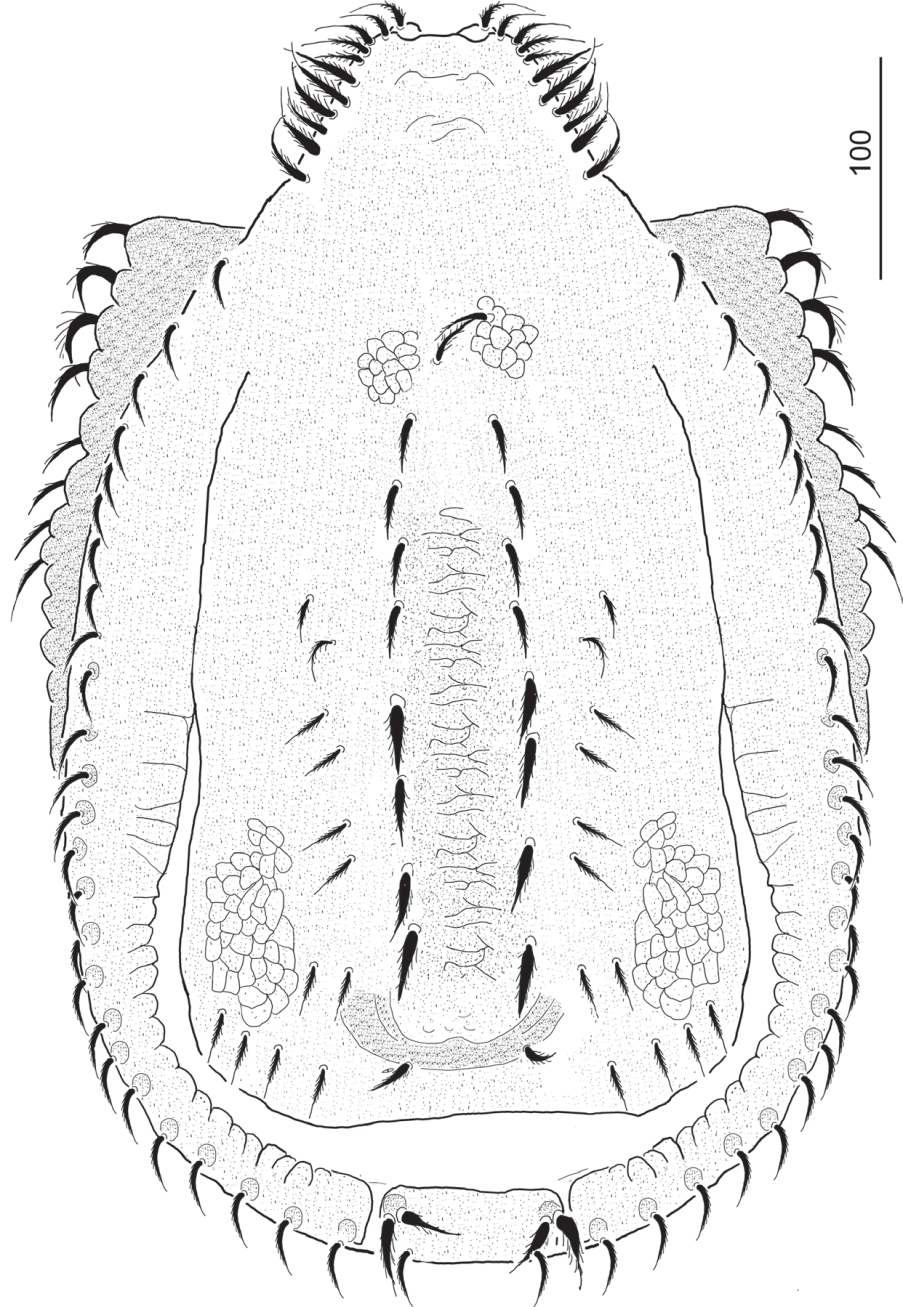
*Ivoriaalourouai* sp. nov., female, holotype, dorsal view.

**Figure 2.**
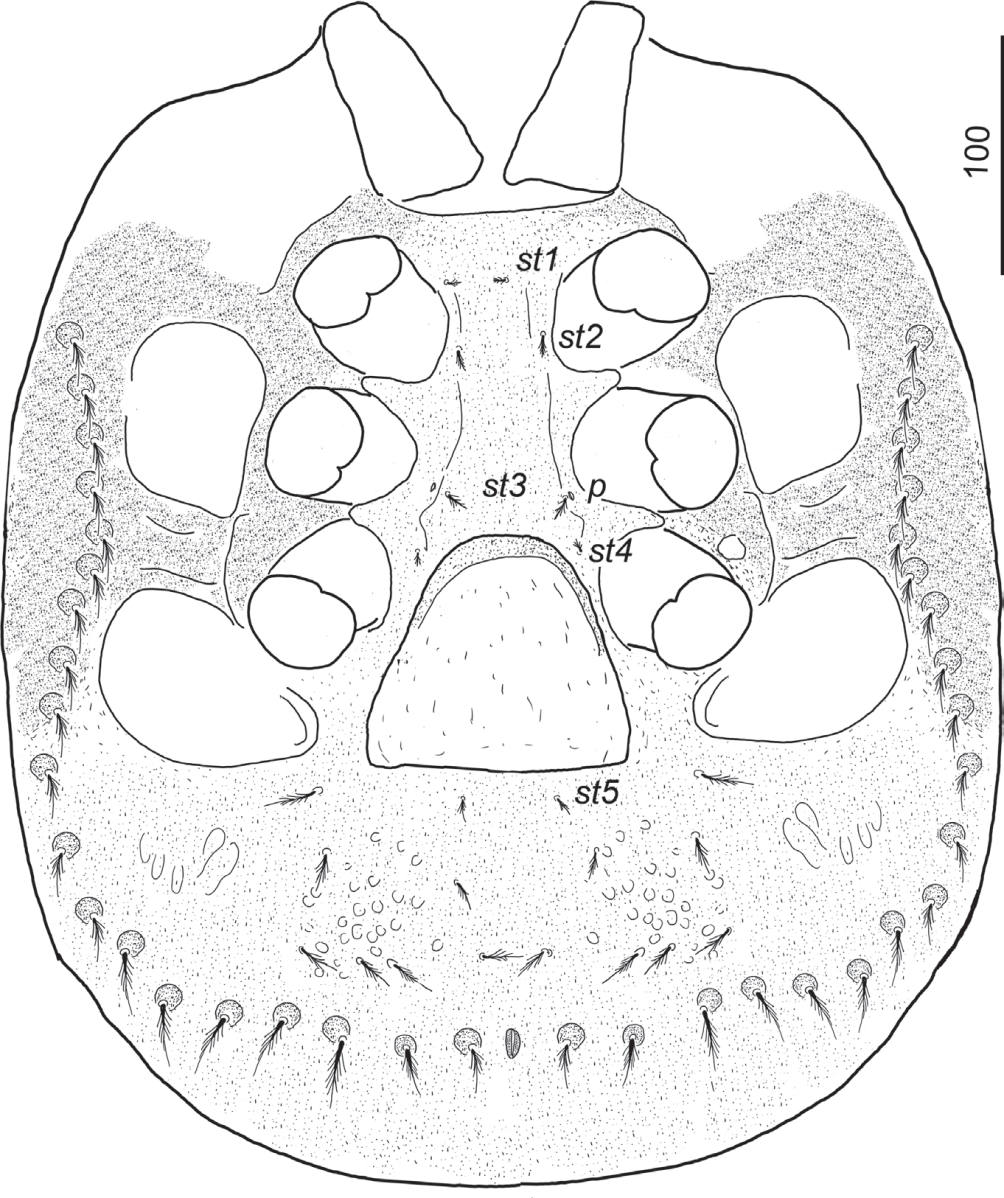
*Ivoriaalourouai* sp. nov., female, holotype, ventral view.

**Figure 3.**
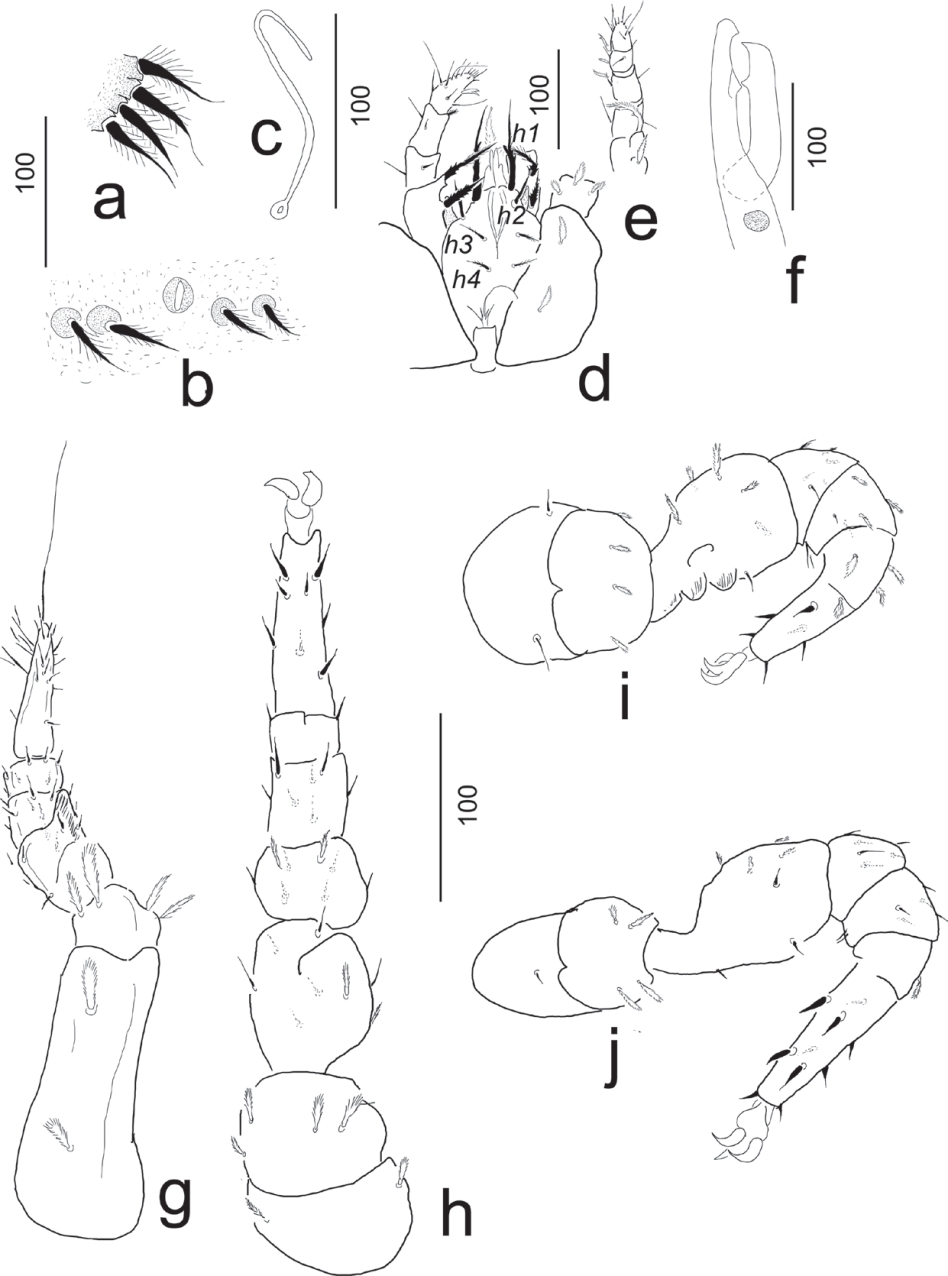
*Ivoriaalourouai* sp. nov., female, holotype. **a** setae on vertex **b** setae around anal opening **c** peritreme **d** ventral view of gnathosoma, coxae I and palp **e** lateral view of palp **f** lateral view of chelicera **g** ventral view of leg I **h** ventral view of leg II **i** lateral view of leg III **j** lateral view of leg IV.

**Figure 4.**
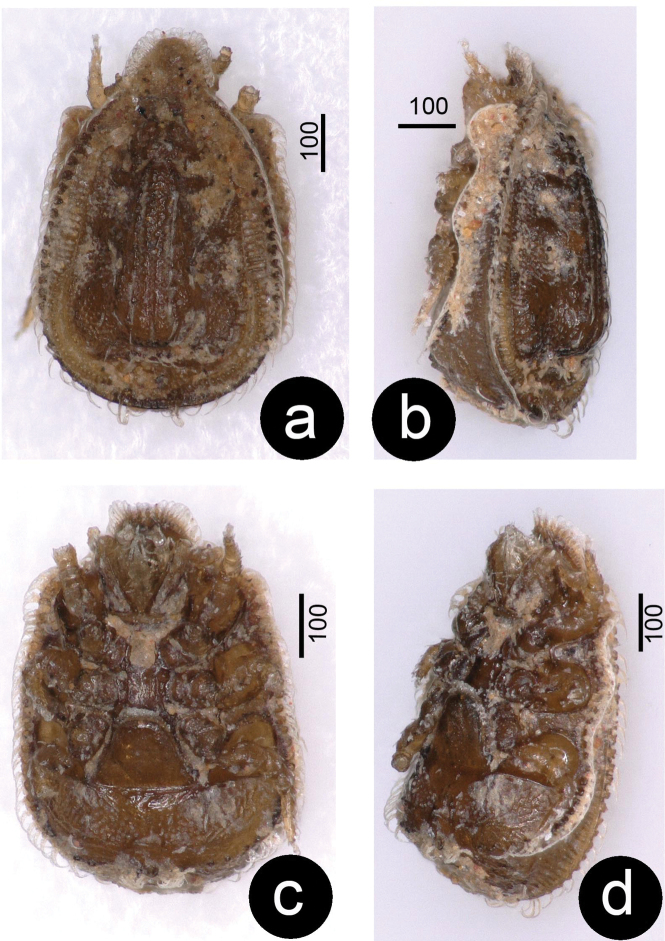
Photos about *Ivoriaalourouai* sp. nov., female, holotype. **a** dorsal view **b** latero-dorsal view **c** ventral view **d** latero-ventral view.

Figures 1 , 2 , 3 , 4

###### Material examined.

***Holotype.*** Female. ”Afrique Occidentale, Côte d’Ivore, Dropleu, tamisage sans tronc mort” (Ivory Coast, Dropleu), 7°24'31"N, 8°19'14"W, 10 Oct. 1980, V. Mahnert and J.L. Peret leg. ***Paratypes.*** Four females, with same collection data as those for the holotype.

###### Diagnosis.

Idiosoma subpentagonal, dorsally domed, marginal and dorsal shields fused anteriorly. All dorsal setae short, with pilose margins. Five pairs of sternal setae pilose. Genital shield of female triangular, anterior margin rounded and situated between coxae IV. Peritreme hook-shaped. Tritosternum with vase-like base, apically serrate, its laciniae subdivided into two pairs of short lateral branches and one pair of long central branches. Hypostomal setae *h1* robust, with a short lateral branch and with numerous lateral teeth, *h2*, *h3*, and *h4* narrow and marginally serrate. Palptrochanter setae *v1* robust and serrate, *v2* situated on small protuberance, basally serrate and apically pilose. Internal malae long and smooth with a short lateral branch.

###### Description.

**Female.** Length of idiosoma 560–580, width at level of coxae IV 360–375 (*N* = 5), color reddish-brown. Shape of idiosoma pentagonal with vertex, dorsally domed.

***Dorsal idiosoma*** (Figs 1, 4a, b). Anterior margin of vertex rounded and margins of vertex bearing marginally pilose setae, ca 32–36 long (Fig. 3a). Marginal and dorsal shields fused anteriorly, dorsal shield elevated on caudal region (Fig. 4b). Majority of dorsal shield covered by reticulate sculptured pattern. Long and robust marginally pilose setae (ca 32–36 long) situated on elevated central area of dorsal shield. 10–12 pairs of short (ca 24–26) and narrow marginally pilose setae situated lateral to elevated area. One pair of poroid situated close to posterior margin of elevated area. Marginal shield without sculptural pattern, inner margins undulate on central and caudal area. All setae on marginal shield ca 23–26 long and marginally pilose. Centro-caudal part of marginal shield separated and forming a quadrangular shield (ca 280–290 wide and ca 30–34 long). This shield bears long and robust marginally pilose setae (ca 32–36) placed on small protuberances.

***Ventral idiosoma*** (Figs 2, 4c, d). Five pairs of sternal setae pilose, *st1* and *st4* shorter (ca 7–8), *st2*, *st3* and *st5* longer (ca 10–12). Setae *st1* inserted at level of anterior margin of coxae II, *st2* at level of central region of coxae II, *st3* at level of posterior margin of coxae III, *st4* at level of central area of coxae III, *st5* close to basal margin of genital shield. Sternal shield without sculptured pattern, one pair of poroid situated close to *st3*. 7–8 pairs of short (ca 21–25), marginally pilose ventral setae situated posterior to genital shield, surface around these setae covered by oval pits. 20–21 pairs of long (ca 34–36), marginally pilose setae placed on small protuberance situated on L-shaped longitudinal row from peritremes to anal opening. Ventral surface bears reticulate sculptural pattern posterior to pedofossae IV. Anal opening oval ca 10–12 long and ca 4–6 wide, anal valves narrow and with smooth surface (Fig. 3b). Genital shield triangular, length 100–105, width at basal level 105–115, situated between coxae IV and pedofossae IV; surface without sculptural pattern. Peritremes hook-shaped (Fig. 3c). Pedofossae deep, their surface smooth, separate furrow for tarsi IV absent. Tritosternum (Fig. 3d) with vase-like base, apically with one pair of spines, its laciniae subdivided into two pairs of short lateral branches and one pair of long central branches.

***Gnathosoma*** (Fig. 3d-f). Corniculi small, smooth and horn-like, situated posterior to *h2*; internal malae smooth with a short lateral branch, two times longer than corniculi. Hypostomal setae *h1* long (ca 90–95), robust and with a short lateral branch and with numerous lateral teeth. Setae *h2* (ca 35–37), *h3* and *h4* (ca 20–24) marginally serrate. Deutosternal region without teeth or denticulate rows. Chelicerae large and robust with internal sclerotized nodes (Fig. 3f). Fixed digit of chelicerae longer (ca 146–150) than movable digit (ca 119–120); both digits of chelicerae bearing a large central tooth. Palp trochanter setae *v1* robust and serrate (ca 49–52), *v2* long (ca 90–92), basally serrate and apically pilose and situated on a small protuberance. Other setae on palp segments smooth (Fig. 3d, e). Palp apotele bifurcated. Epistome marginally serrate.

***Legs*** (Fig. 3g–j). Length of legs (from base of coxae to apex of tarsus): I 340–350, II 380–385, III 330–340, IV 345–355. Leg I without ambulacral claws, majority of setae on all legs pilose, some setae on tarsi and the ventral area smooth.

###### Etymology.

The name of the new species is dedicated to Alouroua the mythical creator of the Akan (Baoule) people who are the major cultural group of the Ivory Coast.

###### Remarks.

Until now, only one species had been described from this poorly-known genus. The differences between of the two species are summarized in Table 1.

**Table 1.** Distinguishing characteristics separating the two known *Ivoria* species.

	*I.taiensis*	*I.alourouai*
Dorsal setae around elevated area	leaf-like with serrate margins	marginally pilose
Centro-caudal part of marginal shield	with two incisions and without separated part	with a separated quadrangular part
Sternal setae	smooth	pilose
Needle-like ventral setae	present	absent
Oval pits on ventral shield	absent	present
Anterior margin of female genital shield	between coxae II	between coxae IV
Shape of anterior margin of female genital shield	peaked	rounded
Peritremes	L-shaped	hook-shaped

## ﻿Discussion

The genus *Ivoria* seems to be a rare, endemic genus in the West African region. The two known species occur only in Ivory Coast. The Uropodina fauna and the distribution of the known species are very poorly investigated in this region, therefore discovery of numerous additional species might be expected. A similar situation exists for the East African Uropodina genus *Bloszykiella* (Kontschán and Ermilov 2020).

## Supplementary Material

XML Treatment for Ivoria

XML Treatment for Ivoria alourouai
